# Analysis of CRISPR-Cas System in *Streptococcus thermophilus* and Its Application

**DOI:** 10.3389/fmicb.2018.00257

**Published:** 2018-02-20

**Authors:** Mengyuan Hao, Yanhua Cui, Xiaojun Qu

**Affiliations:** ^1^Department of Food Science and Engineering, School of Chemistry and Chemical Engineering, Harbin Institute of Technology, Harbin, China; ^2^Institute of Microbiology, Heilongjiang Academy of Sciences, Harbin, China

**Keywords:** *Streptococcus thermophilus*, CRISPR-Cas, gene editing, diversity, model system

## Abstract

CRISPR-Cas (Clustered regularly interspaced short palindromic repeats-CRISPR associated proteins) loci, which provide a specific immunity against exogenous elements, are hypervariable among distinct prokaryotes. Based on previous researches, this review focuses on concluding systematical genome editing protocols in *Streptococcus thermophilus.* Firstly, its protocols and optimized conditions in gene editing are introduced. What’s more, classification and diversity analyses of *S. thermophilus* CRISPR-Cas benefit the further understanding of evolution relationship among *Streptococcus.* Ability of its foreign segment integration and spacer source analyses also indicate a new direction of phage resistance. Above all, all of these point out its potential to be regarded as another model system other than type II CRISPR-Cas in *Streptococcus pyogenes*.

## Introduction

CRISPR-Cas system was found in thermophilic archaea firstly and used to withstand foreign DNA infection (Garneau et al., 2010). It consists of CRISPR and Cas protein, the former is a piece of regular clusters short-interval palindromes, consisting of several repeat–interval sequences; the latter is a kind of proteins associated with the CRISPRs gene, with nuclease and helicase activity (Chylinski et al., 2014). The mechanism of CRISPR-Cas system is similar to RNA interference. Its immune process includes three stages: adaptation, expression and interference (Choi and Lee, 2016). Moreover, a new classification system mainly including three types was proposed by Makarova et al. (2011). Type II CRISPR system has been widely used as an important gene editing tool. It is also called gRNA-Cas9 complex system, since it mainly functions through gRNA-Cas9 compound, which can not only specifically recognize DNA fragments and PAM but also induce cleavage of DNA double strands at target site.

*Streptococcus thermophilus* is one of the most valuable lactic acid bacteria, and widely used in production of dairy products. Besides, its CRISPR/Cas system seems to be another popular research area. After long-term immune and evolution processes, *S. thermophilus* CRISPR/Cas loci present rich diversities. According to the differences of genetic position, repeat sequences and *cas* genes, they are classified into four types, namely CRISPR1, CRISPR2, CRISPR3 and CRISPR4, which are all included in three types of CRISPR-Cas systems. Furthermore, CRISPR sequences vary from strain to strain because of the space amount differences. Hence, this article focus on the diversities of four CRISPR/Cas and CRISPR function researches, such as sgRNA-Cas9 gene editing technology, evolution relationships and anti-phage activity of *S. thermophilus* and its potential of becoming another model creature in Type II CRISPR/Cas researches. This work is of help to understand and accelerate researches about environmental adaptability and anti-phage mechanism of *S. thermophilus*, CRISPR gene editing, comparative genomic studies and evolution of *Streptococcus* genus and even prokaryotes.

## Application of *S. thermophilus* CRISPR-Cas System for Gene Editing

Compared with the extremely low recombineering efficiency, down to 10^-4^–10^-6^ (Sharan et al., 2009), and time-consuming optimization of traditional homologous recombination-based methods, CRISPR system could significantly simplify the mutant construction because of its high editing efficiency (50∼100%) and possibility to select double-crossover events in one step (Chen et al., 2017).

**FIGURE 1:**
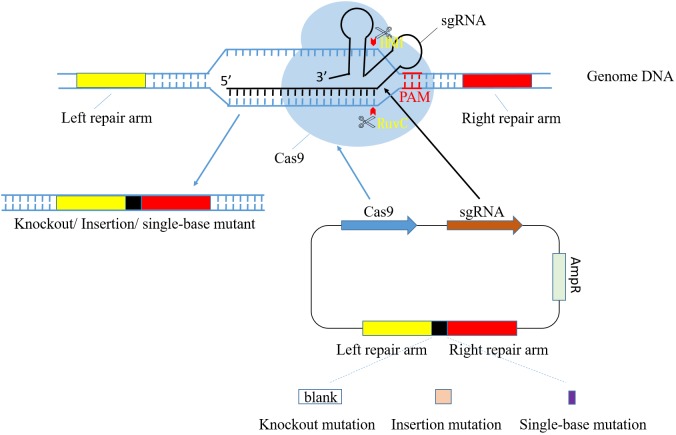
Diagram of CRISPR/Cas9 system-mediated gene editing process. pCas9 plasmid consists of *cas9* gene, sgRNA, resistance locus and homologous arms. Cas9 and sgRNA gene express in order to identify target DNA and cleave at specific sites in front of PAM. RuvC and HNH are both nuclease domain in Cas9, and they cleave target DNA strand and its antisense strand, respectively. Homologous arm sequences in plasmid are complementary to their counterparts in genome so that the impact target gene fragment can be selected accurately. The whole target DNA is able to be deleted, replaced or added with a piece of new sequence through design of different homologous repair arm.

CRISPR-Cas systems start from sequence specific recognition and target DNA loci cleavage and end with fixed mutations through DNA repair mechanism in host cell (Selle and Barrangou, 2015). The process of genome modification mediated by CRISPR-Cas9 is shown in **Figure 1**. Recently, CRISPR-Cas9 system of *S. pyogenes* (SpCas9) has been widely applied as a tool in genome modification. However, shortcomings of this system are noticed as its further study and extensive application (Hsu et al., 2013). Thus, researchers focused on the more progressive *S. thermophilus* CRISPR-Cas9 system (StCas9), which presents lower off-targeting efficiency, improved specificity and smaller Cas9 fragment (Xu et al., 2015; Müller et al., 2016). Its main process includes construction of functional vector containing StCas9 system sequence and expression assay in host cell. Especially, the foremost step is the design of sgRNA, two complementary single-stranded oligo sequences consisting of crRNA and tracrRNA.

Several researches demonstrate that StCas9 shows similar cleavage activity as SpCas9 and feasibility of gene editing in human, plants and microorganisms (Xu et al., 2015; Müller et al., 2016; Steinert et al., 2016). One of the essential parts in StCas9 system is Cas9 protein. As the undertaker of the cutting function, Cas9 is composed of nuclease (NUC) and a recognition (REC) lobe (Anders et al., 2014; Nishimasu et al., 2014). The former, including NUC, RuvC like nuclease and PI domains, is mainly responsible for PAM-specific cutting of target DNA double-strand. And the latter is responsible for recognition of sgRNA-target DNA complex (Chen et al., 2017). StCas9 system includes St1Cas9 and St3Cas9, which represent *S. thermophilus* CRISPR-Cas9 system in CRISPR1 and CRISPR3 loci, respectively. St1Cas9 and St3Cas9 recognize 5′-NNAGAAW and 5′-NGGNG, respectively (Garneau et al., 2010; Magadán et al., 2012). Hence, the target efficiency shown between them is slightly different. St3Cas9 presents higher target efficiency than St1Cas9 (Müller et al., 2016). However, both of them can be used in genome modification according to existing researches (Xu et al., 2015; Müller et al., 2016; Steinert et al., 2016).

Furthermore, there are several pivotal factors affecting activities and editing efficiency of StCas9 system. Firstly, sgRNA, consisting of crRNA (CRISPR RNA) and tracrRNA (*trans*-activating RNA), owns its typical stem-loop structure, which could enhance the interaction between Cas9 and sgRNA (Jinek et al., 2014; Nishimasu et al., 2014). But stem and ring has influence on its activity. For instance, stem-loop structure in 3′ end of tracrRNA plays vital roles in activity of StCas9. And its internal bulges are essential for the functions of StCas9 while the change of side loop sequences has no effect (Xu et al., 2015). Of note, crRNA is the designed spacer acting on target DNA strand to provide specific targeting. tracrRNA is responsible for Cas9 combination and its 3′ region is vital to StCas9 activity (Wiedenheft et al., 2012; Xu et al., 2015). Furthermore the dosage of Cas9 and sgRNA is closely related to off-target efficiency of StCas9 system (Hsu et al., 2013). And truncated tracrRNA could improve its targeting efficiency (Pattanayak et al., 2013). In addition, PAM and its nearby bases are crucial for specific recognition of target fragments. Especially, G in PAM second base is essential for activity of StCas9. In conclusion, StCas9 system can not only be widely used in gene editing but also present more progressive application prospects.

Moreover, a spacer-based *S. thermophilus* CRISPR array assembling method has been reported and its assembling steps have been shown in the previous study (Guo et al., 2015). Thus this can be used as a breakthrough of convenient multiplex targeting (Cong et al., 2013). At first, tracrRNA cassette, *cas9* fragment and CRISPR leader sequence together with CRISPR terminator should be obtained by PCR amplifying. Then amplification products are assembled into a proper vector with particular restriction endonucleases sites. As for multiplex targeting, the strategy of DR (direct repeat)-Spacer guidance is adopted instead of sgRNA guidance (Guo et al., 2015). Proper primers should be synthesized for amplifying of interested sequence containing needed DR arrays and specific spacers firstly. Particularly, when designing primers, consideration of retaining corresponding restriction sites is necessary. Through directly annealing oligonucleotides, the first DR array can be simply assembled into primary CRISPR vector (Xu et al., 2015). Next, primers are used to extend and assemble subsequent spacers and DR sequences into CRISPR locus to form an artificial Spacer-DR structure (Gou et al., 2007). Varieties of specific vectors aiming at different target genes can be reconstructed by this way. Finally, this recombinant plasmid is transferred into recipient cells to obtain target mutants, and phenotype observation and sequencing are carried out to assay its editing efficiency.

Harnessing catalytically inactive Cas9, whose endonucleolytic activity has been eliminated but identification and binding functions are still retained (Qi et al., 2013), together with certain transcription factors preventing or facilitating the banding of RNA polymerase (RNAP) and its promoter, an efficient transcription regulation can be achieved (Bikard et al., 2013). An ideal dCas9 (dead Cas9) could be constructed through mutating D10A and H840A in RuvC and HNH domains respectively (Jinek et al., 2012). Of note, the usual dCas9 is demonstrated to block transcription elongation through competitive inhibition with RNAP, while researchers have converted it into a transcriptional activator by linking it withω subunit of RNAP covalently (Dove and Hochschild, 1998; Bikard et al., 2013). A pool of specific spacers could be assembled into constructed vector simultaneously via Golden Gate assembly. Then, dCas9 system would present a pretty high activation in transcriptional regulation (Chen et al., 2017).

## The Diversity of *S. thermophilus* CRISPR Loci and Their Evolution Relationship

According to CRISPR sequence information of several known genome strains published on CRISPR database^1^, there are more than one type CRISPR loci in most *S. thermophilus* strains. The main four CRISPR types can be distinguished by distinct DR and distribution or composition of *cas* genes. Supplementary Table S1 lists an overview of some basic CRISPR repeat-spacer informations. Moreover, CRISPR-Cas distribution in *S. thermophilus* ND07 is shown in Supplementary Figure S1 and these four loci all belong to known CRISPR-Cas systems.

CRISPR1 and CRISPR3 are attributed to Csn Type II-A and both CRISPR loci play crucial roles in adaptation and interference stages (Magadán et al., 2012). The distribution and architecture of *cas* sequences in CRISPR1 and CRISPR3 loci are relatively conservative, with the same amount of *cas* genes in upstream of their repeat-spacer regions. However, the sequence similarities between them are low, which present 33.6% and 41.3% identity in *cas1* and *cas2*, respectively (Horvath et al., 2008). In addition, they all show strong abilities of new spacers integrating, especially CRISPR1. Several evidences demonstrate this conclusion. Through vitro experiments, *S. thermophilus* can naturally obtain spacers from bacteriophages and plasmids DNA into its CRISPR1 and CRISPR3 (Garneau et al., 2010). And there are more spacers in CRISPR1 locus universally. This characteristic can not only provide intensive identifications of exogenous DNA but also do good to the process of spacer integration. Furthermore, CRISPR1 is most widely distributed in *S. thermophilus*.

CRISPR2/Cas, included in Csm Type III-A system, is far more different in both repeat sequences and distribution of *cas* genes, with 27.78% identity in DR sequences and *cas* genes located at both sides of repeat-spacer region. Besides, as for CRISPR2 locus, high proportion of *cas* but low percentage of repeat-spacer region result in its poor ability of integrating new spacers. Distribution of CRISPR loci shows that CRISPR2 is probably a piece of degenerated fragment originated from gram-positive ancestor (Horvath et al., 2008).

CRISPR4, belonging to Cse Type I-E system, exists only in few strains. With only 28 nt repeat sequences and multiple cas modules distributed upstream the repeat-spacer region, CRISPR4 undergoes an unique transcription process and plays distinct roles during phage resistance. There is no novel spacer addition when foreign DNA invading, but significant up-regulated expression of gene encoding Cascade complex can be observed, especially cas7 (Young et al., 2012). Thus, CRISPR4 mainly functions through antiviral defense mediated by Cascade superfamily. In addition, its *cas2* stop codon coincides with partial sequence of the repeat element (Carte et al., 2014).

It is found that distribution of Cas1 and Cas2 has intensive influences on acquisition of invading sequences. They are able to form Cas1–Cas2-protospacer DNA complex when selecting specific foreign DNA (Wang et al., 2015). And both of them retain among all CRISPR-Cas systems and present conservations, which can be applied in comparative genomic researches (Bolotin et al., 2005). It has been demonstrated that Cas1–Cas2 complex acts as integrase to integrate novel spacers into CRISPR locus (Rollie et al., 2015). In addition, new spacers are prone to integrate into the 5′ end (leading end) of repeat-spacer region, while vestigial spacers are apt to be deleted from its 3′ end (tail end) (Deveau et al., 2008). Thus, the CRISPR tail end exhibits high homologies between different strains while the leading end shows high variability. And the deletion and integration process can occur simultaneously.

Study demonstrated that consistency between CRISPR and Cas protein is relatively high in same type (Horvath et al., 2008). This observation indicates coevolution and cooperation relationships between them. And there are significant homologies of several Cas protein among different species, such as Cas7 and Cas9, which all play major roles in defending foreign DNA (Young et al., 2012). It is found that CRISPR3 is universal in most *Streptococcus* species while CRISPR1 and CRISPR4 exist only in few species (Horvath et al., 2008). Through analysis of spacer sequences and *cas* genes, species exposed to similar surroundings with evolution relationship can be found out.

## Anti-Phage Activity Provided by CRISPR/Cas System

Recently, studies about acquired immunization provided by CRISPR/Cas system imply an effective approach to protect *S. thermophilus* from phages attack (Barrangou et al., 2007; Deveau et al., 2008). *S. thermophilus* phages, belonging to *Siphoviridae* family, have adverse effects on *S. thermophilus* growth and reproduction and lead to huge economic losses in dairy industry consequently. *S. thermophilus* phages are classified into two groups named cos and pac type according to their DNA packaging mechanism (Marrec et al., 1997). DT1, Sfi19, Sfi21 and 7201 belong to cos-type, whereas O1205, Sfi11, and 2972 are pac-type phages (Lévesque et al., 2005). They are all common bacteriophages with complete genome sequenced and present high homologies, which would improve anti-phage ability of CRISPR/Cas system.

The CRISPR-Cas system builds an ‘indestructible wall’ in bacteria, functioning via interfering and cutting off exogenous genes (Choi and Lee, 2016). When upon exposure to phage, host strains integrate short sequences from invading phages as spacers. In addition, new spacer addition is only detected in CRISPR1 and CRISPR3 (Paezespino et al., 2015). And neither CRISPR2 nor CRISPR4 acquired any novel DR sequence although CRISPR4/Cas presented biochemical activities *in vitro* (Sinkunas et al., 2013). After transcription of CRISPR locus containing added DR sequences and further processing, the single stranded crRNA with a second structure combines with Cas or Cascade complex to form CRISPR effect nucleoprotein complex (crRNP). The crRNP functions as endonuclease guided by crRNA. Eventually, the host is immune to the re-invading phage through sequence-specific cleavage of phage DNA.

The homologies between CRISPR spacer sequences and existing sequences in the GenBank database are analyzed (Supplementary Table S2). Firstly, most spacers are homologous with *S. thermophilus* bacteriophages genome. In general, one spacer is homologous with several phages and multiple spacers could resist the same phage. Intriguingly, most spacers in CRISPR2 are homologous with *S. thermophilus* bacteriophage 73, whereas spacers in other CRISPR loci show homologies with several phages, such as bacteriophage Sfi19, Sfi21, 7201, TP-J34 and 20617 etc., especially 20617, which is homologous with the most spacers. And researchers reported that even part of introns coexisting in *S. thermophilus* phages share a putative common ancestor (Lévesque et al., 2005). Secondly, the tail end spacers are inclined to be less homologous with exogenous DNA, so that they play less important roles than the 5′ end one. Similar short homologous fragments are found between *S. thermophilus* bacteriophages and other *Streptococcus* phages. By the way, there are some spacers homologous with plasmids. It is reported that fragments from plasmids are added as spacers in *S. thermophilus* CRISPR loci (Garneau et al., 2010). This provides novel methods to eliminate dissemination of antibiotic-resistance gene.

In addition, researches focusing on coevolution between hosts and phages propose that after exposure to phage invasion, not only do new spacers add to *S. thermophilus* CRISPR loci but also mutations happen in phage genome (Barrangou et al., 2007). And more newly added spacers originate from the coding strand of host genome. It can be presumed that this results from specific PAM motifs, which determine the location of integrated fragments in phage genome. Novel spacers are apt to play more important roles on resisting phages. Of note, spacer deletion has been detected in bacteriophage-insensitive *S. thermophilus* mutants (BIMs) during phage resistance process. And the deletion always occurs at adjacent position with addition, implying possibility that deletion happens on account of homologous recombination between DR sequences (Deveau et al., 2008).

## Potential of Being Another Model System in CRISPR/Cas Researches

*Streptococcus thermophilus* is a common probiotic strain used in diary industry. Researchers focusing on its CRISPR system have established complete and systematic knowledge hierarchy about classification, diversity and evolution of its CRISPR loci (Carte et al., 2014; Wu et al., 2014). Lots of evidences indicate that *S. thermophilus* CRISPR has potential of being a characteristic model in CRISPR-Cas systems. Moreover, *S. thermophilus* is closely related to *S. pyogenes*, whose type II CRISPR/Cas has been regarded as an important model system. *S. thermophilus* CRISPR/Cas9 is used to mediate genome editing in eukaryote (Xu et al., 2015; Fujii et al., 2016). Furthermore, compared to *S. pyogenes*, *S. thermophilus* has higher biosecurities and is readily available (Ferretti et al., 2001).

**FIGURE 2:**
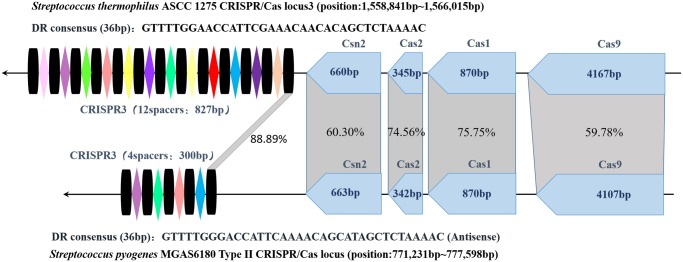
Structure of *S. thermophilus* CRISPR3 and *S. pyogenes* type II CRISPR-Cas loci and their homology comparison. The spacers and repeat sequences are represented by colored rhombi and black columns, respectively. The homology percentage between four Cas protein genes and repeat sequences are indicated in the gray shading. (*S. thermophilus* ASCC 1275 NCBI Reference Sequence: NZ_CP006819.1 and *S. pyogenes* NCBI Reference Sequence: NC_007296.1).

Through homology comparison showed in **Figure 2**, it is found that repeat sequences in *S. thermophilus* CRISPR3 and *S. pyogenes* type II CRISPR-Cas loci share an identity of 88.89% (32/36). And there are much more spacers included in *S. thermophilus* CRISPR3/Cas locus. In addition, both CRISPR-Cas systems show the same structure, with four *cas* genes including *cas9*, *cas1*, *cas2* and *csn2* located upstream and repeat-spacer sequences located downstream the loci. Furthermore, length of each *cas* gene is identical and the four pieces of *cas* sequences present relatively high homology. This is related to their analogous functions. And many studies also demonstrate powerful abilities of *S. thermophilus* type II CRISPR-Cas in integrating exogenous DNA (Deveau et al., 2008). Of note, the common *S. pyogenes* CRISPR system uses NGG as its PAM loci, while *S. themophilus* Cas9 is able to recognize longer PAM. This characteristic imparts St1Cas9 and St3Cas9 with higher targeting efficiency and sequence specificity.

Furthermore there are only two types of CRISPR-Cas loci in most *S. pyogenes* strains genome, including type II and type I-C. Whereas *S. thermophilus* CRISPR-Cas owns more abundant diversities, including Csn type II-A, type I-E and type III-A. In terms of type II-A system, both CRISPR1 and CRISPR3 are endowed with high exogenous DNA resistance activity. Thus, *S. thermophilus* CRISPR-Cas system can not only be another characteristic model but also perform better.

## Conclusion

*Streptococcus thermophilus* CRISPR-Cas system presents rich diversities and excellent abilities of exogenous DNA integration. Plenty of profitable characteristics contribute to its bright application prospects. Owing to the existence of RNA-guided Cas9 nucleases, *S. thermophilus* CRISPR-Cas9 can be used as an efficient genome modification tool with the help of designed sgRNA. In addition, diversity and related evolution relationship provide a unique perspective to classification and evolution researches in *Streptococcus* and other related species. Furthermore, phage infection test *in vitro* together with homology analysis of spacers lay foundation for studies about anti-phage effect of CRISPR-Cas system. Future development of CRISPR-Cas will benefit more from the unfavorable factors that affect its actions and expansion of its application range.

## Author Contributions

YC developed the ideas presented in this manuscript. MH collected the literature and wrote the manuscript. YC and XQ professionally approved the manuscript.

## Conflict of Interest Statement

The authors declare that the research was conducted in the absence of any commercial or financial relationships that could be construed as a potential conflict of interest.

## Footnotes

1 http://crispr.i2bc.paris-saclay.fr
