# Supplementary material for: Analysis of CRISPR-Cas System in Streptococcus thermophilus and Its Application
Source: Front Microbiol. 2018 Feb 20;9:257. doi: 10.3389/fmicb.2018.00257 (PMC5826314; doi:10.3389/fmicb.2018.00257)
Supplement: Supplementary file 1 [file Image_1.pdf]

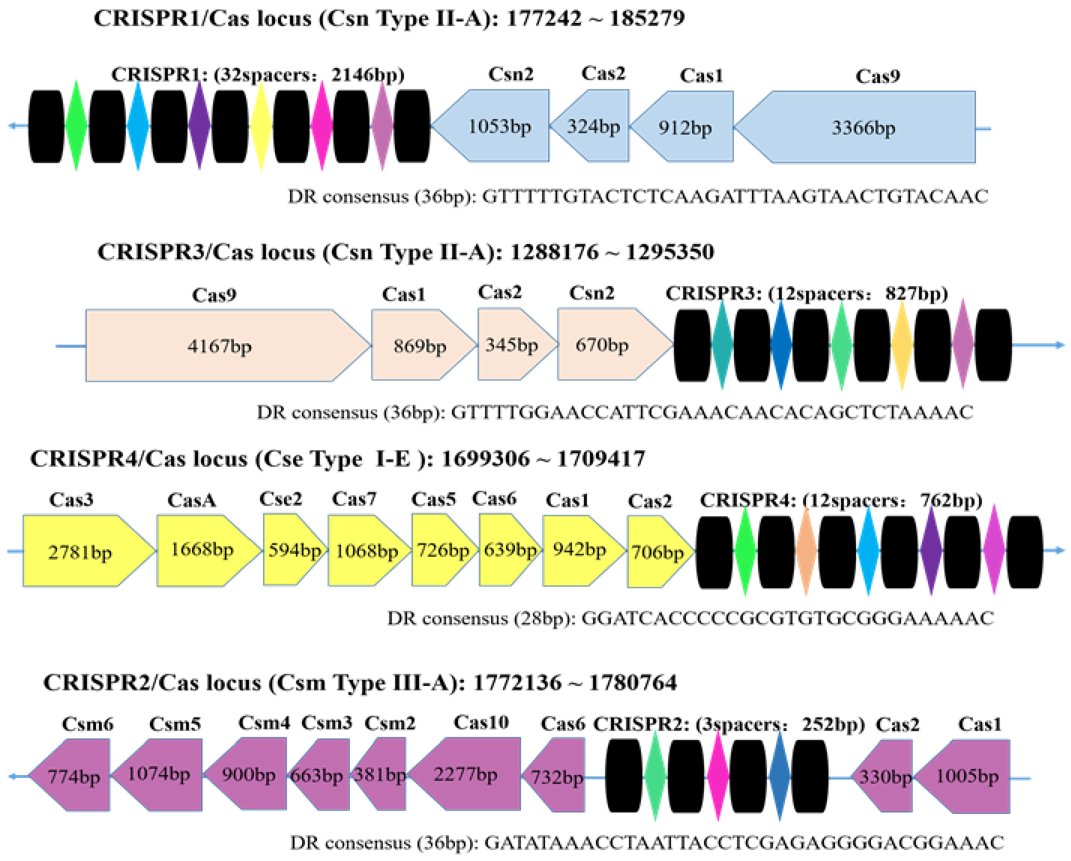

FIGURE S1| Structure of CRISPR/Cas loci in *S. thermophilus* ND07. DRs and spacers are marked with black columns and colored rhombi, respectively. Each pentagon represents a piece of *cas* gene. The order of CRISPR/Cas loci is sorted by their positions in *S. thermophilus* ND07 genome. (NCBI Reference Sequence: NZ_CP016394.1)

TABLE S1 Information of *S. thermophilus* CRISPR Sequences

Strain	CRISPR Type	CRISPR_id	Start Position	End Position	Number of spacers	DR Consensus
ND07	CRISPR1	NZ_CP016394_1	177242	179388	32	GTTTTTGTACTCTCAAGATTTAAGTAACTGTACAAC
	CRISPR3	NZ_CP016394_2	1294523	1295350	12	GTTTTGGAACCATTCGAAACAACACAGCTCTAAAAC
	CRISPR4	NZ_CP016394_3	1708655	1709417	12	GGATCACCCCCGCGTGTGCGGGAAAAAC
	CRISPR2	NZ_CP016394_4	1779078	1779330	3	GATATAAACCTAATTACCTCGAGAGGGGACGGAAAC
ASCC 1275	CRISPR1	NZ_CP006819_4	823246	825392	32	GTTTTTGTACTCTCAAGATTTAAGTAACTGTACAAC
	CRISPR2	NZ_CP006819_5	1074866	1075124	3	GATATAAACCTAATTACCTCGAGAGGGGACGGAAAC
	CRISPR4	NZ_CP006819_6	1144778	1145540	12	GGATCACCCCCGCGTGTGCGGGAAAAAC
	CRISPR3	NZ_CP006819_7	1558841	1559668	12	GTTTTGGAACCATTCGAAACAACACAGCTCTAAAAC
JIM 8232	CRISPR1	NC_017581_1	712332	715137	42	GTTTTTGTACTCTCAAGATTTAAGTAACTGTACAAC
	CRISPR2	NC_017581_2	969817	971087	17	GATATAAACCTAATTACCTCGAGAGGGGACGGAAAC
	CRISPR3	NC_017581_3	1452282	1452514	3	GTTTTGGAACCATTCGAAACAACACAGCTCTAAAAC
LMD-9	CRISPR1	NC_008532_2	649125	650217	16	GTTTTTGTACTCTCAAGATTTAAGTAACTGTACAAC
	CRISPR2	NC_008532_4	897070	897328	3	GATATAAACCTAATTACCTCGAGAGGGGACGGAAAC
	CRISPR3	NC_008532_5	1377229	1377794	8	GTTTTGGAACCATTCGAAACAACACAGCTCTAAAAC
MN-ZLW-002	CRISPR1	NC_017927_1	630165	632177	30	GTTTTTGTACTCTCAAGATTTAAGTAACTGTACAAC
	CRISPR2	NC_017927_2	905670	905771	1	GATATAAACCTAATTACCTCGAGAGGGGACGGAAAC
	CRISPR3	NC_017927_3	1372720	1374472	26	GTTTTGGAACCATTCGAAACAACACAGCTCTAAAAC
ND03	CRISPR1	NC_017563_1	639512	641921	36	GTTTTTGTACTCTCAAGATTTAAGTAACTGTACAAC
	CRISPR2	NC_017563_2	873997	874098	1	GATATAAACCTAATTACCTCGAGAGGGGACGGAAAC
	CRISPR3	NC_017563_3	1363057	1364415	20	GTTTTGGAACCATTCGAAACAACACAGCTCTAAAAC
LMG 18311	CRISPR1	NC_006448_1	629901	632113	33	GTTTTTGTACTCTCAAGATTTAAGTAACTGTACAAC
	CRISPR2	NC_006448_2	863999	864323	4	GATATAAACCTAATTACCTCGAGAGGGGACGGAAAC
CNRZ1066	CRISPR1	NC_006449_1	625101	627844	41	GTTTTTGTACTCTCAAGATTTAAGTAACTGTACAAC

TABLE S2 Summary of homologous relationship between spacers and other sequences.

CRISPR Type	Strain	Spacer	Homologous gene																								
			Phage																						plasmid		
			S. thermophilus bacteriophage																					*S. salivarius* bacteriophage			
			Sfi19	Sfi21	DT1.1	7201	DT1	O1205	73	CHPC926	CHPC1511	CHPC577	TP-778L	TP-J34	53	20617	Abc2	ALQ13.2	128	5093	9871	2972	9874	YMC-2011	pSt08	pSt106	pND103
			AF115102.1	AF115103.1	AF348744.1	AF145054.1	AF085222.2	U88974.1	KT717083.1	KX879642.1	KX879643.1	KX879641.1	HG380752.1	HE861935.1	KT717084.1	HG424323.1	FJ236310.1	FJ226752.1	KT717085.1	FJ965538.1	KU678389.	AY699705.	KU678392.	CP002889.1	AJ239049.2	AJ242479.2	AY250830.1
		Spacer1	93%	93%	—	—	—	—	—	—	—	—	—	—	—	—	—	—	—	—	—	—	—	—	—	—	—
		Spacer2	—	—	—	—	—	—	—	—	—	—	—	—	—	100%	—	—	—	—	—	—	—	—	—	—	—
		Spacer3	100%	—	—	—	—	—	—	—	—	—	—	—	—	—	—	—	—	—	—	—	—	—	—	—	—
		Spacer4*	—	—	—	—	—	—	—	—	—	—	—	—	—	—	—	—	—	—	—	—	—	—	—	—	—
		Spacer5	100%	—	—	—	—	—	—	—	—	—	—	—	—	—	—	—	—	—	—	—	—	—	—	—	—
		Spacer6*	—	—	—	—	—	—	—	—	—	—	—	—	—	—	—	—	—	—	—	—	—	—	—	—	—
		Spacer7*	—	—	—	—	—	—	—	—	—	—	—	—	—	—	—	—	—	—	—	—	—	—	—	—	—
		Spacer8	—	—	—	100%	—	—	—	—	—	—	—	—	97%	—	—	—	97%	—	—	—	—	—	—	—	—
		Spacer9	—	—	—	—	—	—	—	—	—	—	—	100%	—	100%	—	—	—	100%	—	—	—	—	—	—	—
		Spacer10*	—	—	—	—	—	—	—	—	—	—	—	—	—	—	—	—	—	—	—	—	—	—	—	—	—
		Spacer11	—	—	—	—	—	—	—	—	—	—	—	—	—	100%	—	—	—	—	—	—	—	—	—	—	—
		Spacer12	—	—	—	—	—	—	—	—	—	—	—	—	—	100%	—	—	—	—	—	—	—	—	—	—	—
		Spacer13	—	—	—	100%	—	—	—	—	—	—	—	—	—	—	—	—	—	—	—	—	100%	—	—	—	—
	ND07	Spacer14	—	—	—	100%	—	—	—	—	—	—	—	—	—	100%	—	—	—	—	—	—	—	—	—	—	—
CRISPR1		Spacer15	—	97%	—	—	—	—	—	—	—	—	—	—	—	—	—	—	—	—	—	—	—	97%	—	—	—
		Spacer16*	—	—	—	—	—	—	—	—	—	—	—	—	—	—	—	—	—	—	—	—	—	—	—	—	—
		Spacer17	—	—	—	—	—	—	—	—	—	—	—	—	—	100%	—	—	—	—	—	—	—	—	—	—	—
		Spacer18	—	—	—	—	—	—	—	—	—	—	—	—	—	—	—	—	—	—	—	—	100%	—	—	—	—
		Spacer19	—	—	—	—	—	—	—	—	—	—	—	—	—	—	—	—	—	—	—	—	100%	96%	—	—	—
		Spacer20	—	—	—	—	100%	—	—	—	—	100%	—	—	100%	—	—	—	100%	—	—	—	—	—	—	—	—
		Spacer21	—	—	—	—	—	—	—	—	100%	—	—	100%	—	100%	—	—	—	100%	—	—	—	—	—	—	—
		Spacer22	—	—	—	—	—	—	—	100%	—	—	—	—	—	—	—	—	—	—	—	—	—	—	—	—	—
		Spacer23	—	—	—	—	—	—	—	—	—	—	—	—	—	100%	—	—	—	—	—	—	—	—	—	—	—
		Spacer24*	—	—	—	—	—	—	—	—	—	—	—	—	—	—	—	—	—	—	—	—	—	—	—	—	—
		Spacer25	—	—	—	—	—	—	—	—	—	—	—	—	—	100%	—	—	—	—	—	—	—	—	—	—	—
		Spacer26*	—	—	—	—	—	—	—	—	—	—	—	—	—	—	—	—	—	—	—	—	—	—	—	—	—
		Spacer27*	—	—	—	—	—	—	—	—	—	—	—	—	—	—	—	—	—	—	—	—	—	—	—	—	—
		Spacer28*	—	—	—	—	—	—	—	—	—	—	—	—	—	—	—	—	—	—	—	—	—	—	—	—	—
		Spacer29*	—	—	—	—	—	—	—	—	—	—	—	—	—	—	—	—	—	—	—	—	—	—	—	—	—
		Spacer30*	—	—	—	—	—	—	—	—	—	—	—	—	—	—	—	—	—	—	—	—	—	—	—	—	—
		Spacer31	—	—	—	—	—	—	—	—	—	—	—	—	—	—	97%	97%	—	—	—	—	—	—	—	—	—
		Spacer32*	—	—	—	—	—	—	—	—	—	—	—	—	—	—	—	—	—	—	—	—	—	—	—	—	—
	LMD-9	Spacer1*	—	—	—	—	—	—	—	—	—	—	—	—	—	—	—	—	—	—	—	—	—	—	—	—	—
		Spacer2	—	—	—	100%	—	—	—	—	—	—	—	—	—	—	—	—	—	—	—	—	—	—	—	—	—

		Spacer3	—	—	—	—	—	—	—	—	—	—	100%	—	—	—	—	—	—	—	—	—	—	—	—	—	—
		Spacer4	—	—	—	—	—	—	—	—	—	—	—	—	—	—	—	—	100%	—	—	—	—	—	—	—	—
		Spacer5	—	—	100%	—	—	—	—	—	—	—	—	—	—	—	—	—	—	—	—	—	—	—	—	—	—
		Spacer6	—	—	—	—	—	—	—	—	—	—	—	—	—	—	—	—	—	—	—	—	—	—	100%	—	—
		Spacer7*	—	—	—	—	—	—	—	—	—	—	—	—	—	—	—	—	—	—	—	—	—	—	—	—	—
		Spacer8*	—	—	—	—	—	—	—	—	—	—	—	—	—	—	—	—	—	—	—	—	—	—	—	—	—
		Spacer9*	—	—	—	—	—	—	—	—	—	—	—	—	—	—	—	—	—	—	—	—	—	—	—	—	—
		Spacer10*	—	—	—	—	—	—	—	—	—	—	—	—	—	—	—	—	—	—	—	—	—	—	—	—	—
		Spacer11*	—	—	—	—	—	—	—	—	—	—	—	—	—	—	—	—	—	—	—	—	—	—	—	—	—
		Spacer12*	—	—	—	—	—	—	—	—	—	—	—	—	—	—	—	—	—	—	—	—	—	—	—	—	—
		Spacer13	100%	—	—	—	—	—	—	—	—	—	—	—	—	—	—	—	—	—	—	—	—	—	—	—	—
		Spacer14*	—	—	—	—	—	—	—	—	—	—	—	—	—	—	—	—	—	—	—	—	—	—	—	—	—
		Spacer15*	—	—	—	—	—	—	—	—	—	—	—	—	—	—	—	—	—	—	—	—	—	—	—	—	—
		Spacer16	—	—	—	—	—	—	—	—	—	—	100%	—	—	—	—	—	—	—	—	—	—	—	—	—	—
	JIM—8232	Spacer1*	—	—	—	—	—	—	—	—	—	—	—	—	—	—	—	—	—	—	—	—	—	—	—	—	—
		Spacer2	—	—	—	100%	—	—	—	—	—	—	—	—	—	—	—	—	—	—	—	—	—	—	—	—	—
		Spacer3	—	—	—	—	—	—	—	—	—	—	—	—	—	—	—	—	—	100%	—	—	—	—	—	—	—
		Spacer4	—	—	—	—	—	—	—	—	—	—	—	—	—	100%	—	—	—	—	—	—	—	—	—	—	—
		Spacer5	—	—	—	—	—	—	—	—	—	—	—	—	—	—	—	—	—	—	—	—	—	—	93%	—	—
		Spacer6	—	—	—	100%	—	—	—	—	—	—	—	—	—	—	—	—	—	—	—	—	—	—	—	—	—
		Spacer7	—	—	—	—	—	—	—	—	—	—	—	—	—	—	—	—	—	—	—	—	—	—	—	93%	—
		Spacer8	—	—	—	—	—	—	—	—	—	96%	—	—	—	—	—	—	—	—	—	—	—	—	—	—	—
		Spacer9*	—	—	—	—	—	—	—	—	—	—	—	—	—	—	—	—	—	—	—	—	—	—	—	—	—
		Spacer10*	—	—	—	—	—	—	—	—	—	—	—	—	—	—	—	—	—	—	—	—	—	—	—	—	—
		Spacer11*	—	—	—	—	—	—	—	—	—	—	—	—	—	—	—	—	—	—	—	—	—	—	—	—	—
		Spacer12	—	—	—	—	—	—	—	—	—	—	—	—	100%	100%	—	100%	—	—	—	—	—	—	—	—	—
		Spacer13	—	—	—	—	—	—	—	—	100%	—	—	—	—	—	—	—	—	—	—	—	—	—	—	—	—
		Spacer14*	—	—	—	—	—	—	—	—	—	—	—	—	—	—	—	—	—	—	—	—	—	—	—	—	—
		Spacer15	—	—	—	—	—	—	—	—	—	—	—	—	100%	—	100%	100%	—	—	—	—	—	—	—	—	—
		Spacer16	—	—	—	—	—	—	—	—	—	—	—	—	—	96%	—	—	—	—	—	—	—	—	—	—	—
		Spacer17	—	—	—	—	100%	—	100%	—	—	—	—	—	100%	—	100%	—	100%	—	—	—	—	—	—	—	—
		Spacer18	—	—	—	—	—	—	100%	—	—	—	—	—	100%	—	—	—	100%	—	—	—	—	—	—	—	—
		Spacer19	—	—	—	100%	—	—	—	—	—	—	—	—	—	—	—	—	—	—	—	—	—	—	—	—	—
		Spacer20	—	—	—	—	—	—	—	—	—	—	—	—	—	—	—	—	—	—	—	—	—	—	—	—	—
		Spacer21	—	—	—	100%	100%	—	—	—	—	—	—	—	—	—	—	97%	97%	—	—	—	—	—	—	—	—
		Spacer22	—	—	—	—	—	—	—	—	—	—	—	—	—	—	—	—	—	—	—	—	—	—	—	—	—
		Spacer23	—	—	—	100%	—	—	—	—	—	—	—	—	—	—	—	—	—	—	—	—	—	—	—	—	—
		Spacer24	—	100%	—	100%	—	—	—	—	—	—	—	—	—	—	—	—	—	—	—	—	—	—	—	—	—
		Spacer25	—	—	—	—	—	—	—	—	—	—	—	—	—	—	—	—	—	—	—	—	—	—	—	93%	—
		Spacer26	—	—	—	—	—	—	—	—	—	96%	—	—	—	—	—	—	—	—	—	—	—	—	—	—	—
		Spacer27	—	—	—	—	—	—	100%	—	—	—	—	—	—	—	100%	—	—	—	—	—	—	—	—	—	—
		Spacer28	—	—	—	—	—	—	—	—	—	—	—	—	—	100%	100%	—	—	—	—	—	—	—	—	—	—

		Spacer29	—	—	—	100%	—	—	—	—	—	—	—	—	—	—	—	—	—	—	—	—	—	—	—	—	—
		Spacer30	—	—	—	100%	—	—	—	—	—	—	—	—	—	—	—	—	—	—	—	—	—	—	—	—	—
		Spacer31*	—	—	—	—	—	—	—	—	—	—	—	—	—	—	—	—	—	—	—	—	—	—	—	—	—
		Spacer32	—	—	—	100%	—	—	—	—	—	—	—	—	—	100%	—	—	—	—	—	—	—	—	—	—	—
		Spacer33	—	—	—	—	—	—	—	—	97%	—	—	—	—	—	—	—	—	—	—	—	—	—	—	—	—
		Spacer34	—	—	—	—	—	—	—	—	100%	—	—	—	—	—	—	—	—	100%	—	—	—	—	—	—	—
		Spacer35*	—	—	—	—	—	—	—	—	—	—	—	—	—	—	—	—	—	—	—	—	—	—	—	—	—
		Spacer36*	—	—	—	—	—	—	—	—	—	—	—	—	—	—	—	—	—	—	—	—	—	—	—	—	—
		Spacer37	—	—	—	—	—	—	—	—	—	—	—	—	100%	—	100%	100%	—	—	—	—	—	—	—	—	—
		Spacer38*	—	—	—	—	—	—	—	—	—	—	—	—	—	—	—	—	—	—	—	—	—	—	—	—	—
		Spacer39*	—	—	—	—	—	—	—	—	—	—	—	—	—	—	—	—	—	—	—	—	—	—	—	—	—
		Spacer40	—	—	—	—	96%	—	—	—	—	—	—	—	—	—	—	—	—	—	—	—	—	—	—	—	—
		Spacer41*	—	—	—	—	—	—	—	—	—	—	—	—	—	—	—	—	—	—	—	—	—	—	—	—	—
		Spacer42*	—	—	—	—	—	—	—	—	—	—	—	—	—	—	—	—	—	—	—	—	—	—	—	—	—
CRISPR3	ND07	Spacer1	97%	—	—	100%	—	—	—	—	—	—	—	—	—	—	—	—	—	—	—	—	—	—	—	—	—
		Spacer2	—	—	—	—	—	—	—	—	—	—	—	—	—	100%	—	—	—	—	—	—	100%	—	—	—	—
		Spacer3*	—	—	—	—	—	—	—	—	—	—	—	—	—	—	—	—	—	—	—	—	—	—	—	—	—
		Spacer4	—	—	—	—	—	—	—	—	—	—	—	—	—	100%	—	—	—	—	100%	—	—	—	—	—	—
		Spacer5	—	—	—	—	—	—	—	—	—	100%	—	—	—	—	—	—	—	—	—	—	100%	—	—	—	—
		Spacer6*	—	—	—	—	—	—	—	—	—	—	—	—	—	—	—	—	—	—	—	—	—	—	—	—	—
		spacer7	—	—	—	—	100%	—	—	—	—	—	—	—	—	—	—	100%	100%	—	—	100%	—	—	—	—	—
		Spacer8	—	—	—	—	—	—	—	—	—	—	—	—	—	—	—	—	—	—	—	—	—	96%	—	—	—
		Spacer9*	—	—	—	—	—	—	—	—	—	—	—	—	—	—	—	—	—	—	—	—	—	—	—	—	—
		Spacer10	—	—	—	—	—	—	—	—	—	—	96%	96%	—	—	—	—	—	—	—	—	—	—	—	—	—
		Spacer11	—	—	—	—	—	—	—	—	—	—	—	—	—	100%	—	—	—	—	100%	—	—	—	—	—	—
		Spacer12	—	—	—	—	—	—	—	—	—	—	100%	—	—	—	—	—	—	—	100%	100%	100%	—	—	—	—
	LMD-9	Spacer1	100%	—	—	—	—	—	—	—	—	—	—	—	—	—	—	—	100%	—	—	—	—	—	—	—	—
		Spacer2*	—	—	—	—	—	—	—	—	—	—	—	—	—	—	—	—	—	—	—	—	—	—	—	—	—
		Spacer3	100%	—	—	—	—	—	—	—	—	—	—	—	—	—	—	—	100%	—	—	—	—	—	—	—	—
		Spacer4	—	—	—	—	—	—	—	—	—	—	—	100%	—	—	—	—	—	—	—	—	—	—	—	—	—
		Spacer5	—	—	—	—	—	—	—	—	—	—	—	—	—	—	—	—	100%	—	—	—	—	—	—	—	—
		Spacer6	—	—	—	—	—	—	—	—	—	—	—	—	—	—	—	—	—	—	—	—	—	—	—	—	100%
		Spacer7	—	—	—	—	—	—	—	—	—	—	—	—	—	—	—	—	—	—	—	—	—	—	—	100%	—
		Spacer8*	—	—	—	—	—	—	—	—	—	—	—	—	—	—	—	—	—	—	—	—	—	—	—	—	—
CRISPR4	ND07	Spacer1	—	—	—	—	—	—	—	—	—	—	—	—	—	—	—	97%	—	—	—	—	—	—	—	—	—
		Spacer2*	—	—	—	—	—	—	—	—	—	—	—	—	—	—	—	—	—	—	—	—	—	—	—	—	—
		Spacer3	—	—	—	—	—	—	—	—	—	—	100%	—	—	100%	—	—	—	—	—	—	—	—	—	—	—
		Spacer4	—	—	—	—	—	—	—	—	—	—	—	—	—	100%	—	—	—	—	—	—	—	—	—	—	—
		Spacer5	—	—	—	—	—	—	—	—	—	—	—	—	—	—	—	97%	—	—	—	—	—	—	—	—	—
		Spacer6	97%	97%	—	—	—	97%	—	—	—	—	—	—	—	—	—	—	—	—	—	—	—	—	—	—	—
		Spacer7	100%	100%	—	—	—	—	—	—	—	—	—	—	—	—	—	—	—	—	—	—	—	—	—	—	—
		Spacer8	97%	97%	—	—	—	—	—	—	—	—	—	—	—	94%	—	—	—	—	—	—	—	—	—	—	—

		Spacer9	—	—	—	—	—	—	—	—	—	—	94%	94%	—	—	—	—	—	—	—	—	—	—	—	—	—
		Spacer10	—	—	—	100%	—	—	—	—	—	—	—	—	—	—	—	—	100%	—	—	—	—	—	—	—	—
		Spacer11*	—	—	—	—	—	—	—	—	—	—	—	—	—	—	—	—	—	—	—	—	—	—	—	—	—
		Spacer12*	—	—	—	—	—	—	—	—	—	—	—	—	—	—	—	—	—	—	—	—	—	—	—	—	—
CRISPR2	ND07	Spacer1	—	—	—	—	100%	—	100%	—	—	—	—	—	—	—	—	—	—	—	—	—	—	—	—	—	—
		Spacer2	—	—	—	—	—	—	94%	—	—	—	—	—	—	—	—	—	—	—	—	—	—	—	—	—	—
		Spacer3*	—	—	—	—	—	—	—	—	—	—	—	—	—	—	—	—	—	—	—	—	—	—	—	—	—
	LMD-9	Spacer1	—	—	—	—	—	—	—	—	—	—	—	—	—	—	—	—	—	—	—	—	—	—	—	—	—
		Spacer2	—	—	—	—	—	—	94%	—	—	—	—	—	—	—	—	—	—	—	—	—	—	—	—	—	—
		Spacer3	—	—	—	—	97%	—	97%	—	—	—	—	—	—	—	—	—	—	—	—	—	—	—	—	—	—
	JIM—8232	Spacer1*	—	—	—	—	—	—	—	—	—	—	—	—	—	—	—	—	—	—	—	—	—	—	—	—	—
		Spacer2*	—	—	—	—	—	—	—	—	—	—	—	—	—	—	—	—	—	—	—	—	—	—	—	—	—
		Spacer3*	—	—	—	—	—	—	—	—	—	—	—	—	—	—	—	—	—	—	—	—	—	—	—	—	—

* Spacers not homologous with exogenous sequences.

— Not homologous to the exogenous DNA.
